# Fertility-sparing surgery for young patients with borderline ovarian tumors (BOTs): single institution experience

**DOI:** 10.1186/s13048-016-0226-y

**Published:** 2016-03-18

**Authors:** Rui-fang Chen, Jun Li, Ting-ting Zhu, Hai-lin Yu, Xin Lu

**Affiliations:** Department of Gynecologic Oncology, the Obstetrics and Gynecology Hospital of Fudan University, 128# Shen-yang Road in Yangpu District, Shanghai, 200000 China

**Keywords:** Borderline ovarian tumors (BOTs), Fertility sparing surgery, Surgical procedure, Surgical approach, Staging, Chemotherapy, Recurrence, Pregnancy

## Abstract

**Background:**

Fertility-sparing surgery for patients with borderline ovarian tumors (BOTs) is still controversial. This study aimed to evaluate the oncological safety and fertility benefits in conservative surgery,as well as efficiency of surgical procedures and approaches.

**Results:**

In total 122 patients with BOTs, four types of fertility-sparing surgery were performed: unilateral adnexectomy (UA, *n* = 47), unilateral cystectomy (UC, *n* = 59), unilateral adnexectomy + contralateral cystectomy (UA + CC, *n* = 7) and bilateral cystectomy (BC, *n* = 9). Fifty-two (42.6 %) patients had undergone laparoscopy, while 70 (57.4 %) had undergone laparotomy. After a median follow-up of 58.0 months, eight patients (6.6 %) relapsed in average of 25.9 months. Only one patient progressed to invasive cancer. None died within our observational period. Univariate analysis showed that patients with elevated CA125, bilateral tumors, extra-ovary tumor or mucinous type tended to replase in shorter time (*p* < 0.05). Among all cases, 45 patients attempted to conceive and 34 (75.6 %) patients had successful pregnancy.

The recurrence rates were successively increased (2.1 %, 6.8 %, 14.3 %, and 22.2 %), the recurrence interval were shortened (48.0, 25.3, 26.0 and 21.2 months) and the subsequent fertility rates were 76.9 %, 77.3 %, 66.7 % and 71.4 % in UA, UC, UA + CC, and BC groups, respectively. As for surgical approaches, three patients (5.8 %) relapsed in 26.3 months in the laparoscopy group and five (7.1 %) in 25.5 months in the laparotomy group. The subsequent fertility rate was higher in laparoscopy group (88.9 %) than in laparotomy group (66.7 %).

In our study, 38 patients underwent staging surgery. Two patients (5.3 %) recurrent in average of 21.0 months, and the subsequent pregnancy rate of staging surgery group was 61.5 %. Twelve patients received adjuvant chemotherapy but they didn’t get any benefit from it, both in term of recurrence (8.3 %, 26.0 months) and subsequent pregnancy rate (75.5 %).

**Conclusion:**

Fertility-sparing surgery is safe and beneficial for most young BOTs. UA through laparoscopy should be recommended as the first choice. To the patients with bilateral tumors, elevated CA125, extra-ovary tumor or mucinous type, conservative surgery should be carefully chosen and subsequent pregnancy should be attempted in short term. In addition, the benefit of comprehensive surgical staging is to be further investigated and adjuvant chemotherapy is not recommended.

## Background

Borderline ovarian tumors (BOTs) are defined as an epithelial ovarian tumor exhibiting an atypical epithelial proliferation without destructive stromal invasion. They account for 10–20 % of all ovarian epithelial tumors, with an incidence of 1.8–4.8 per 100,000 women per year [1, 2]. The International Federation of Gynecology and Obstetrics (FIGO) acknowledged BOTs as a separate entity in 1961 and the World Health Organization (WHO) recognized the classification in 1973. Three terms are currently used to refer these tumors: borderline tumor, tumor of low malignant potential, and atypical proliferative tumor. Compared to invasive epithelial ovarian cancers, BOTs are typically present in younger women, primarily diagnosed at earlier stages, and result in more favorable prognosis [3, 4]. Over the past several decades, the incidence of BOT has been increased, and surgical therapy has shifted from a radical approach to more conservative treatment. Recent studies have indicated that the fertility-sparing treatment with BOTs is well tolerated and able to permit future pregnancy [5, 6]. However, the fertility sparing treatment may increase the risk of relapse and sometimes lead to death [7–9]. Therefore, oncological safety must always be balanced at the same time. There was no randomized clinical trial to concern the prognostic factors of recurrence and subsequent fertility rate in different fertility sparing therapeutic strategies. Therefore, the optimal clinical strategies for young patients with BOT are often discussed. The purpose of this study is to analyze the oncological safety of fertility-sparing surgery, the efficacy of different surgical procedures and approaches, as well as adjuvant chemotherapy on the recurrence rate and reproductive outcomes.

**Fig 1:**
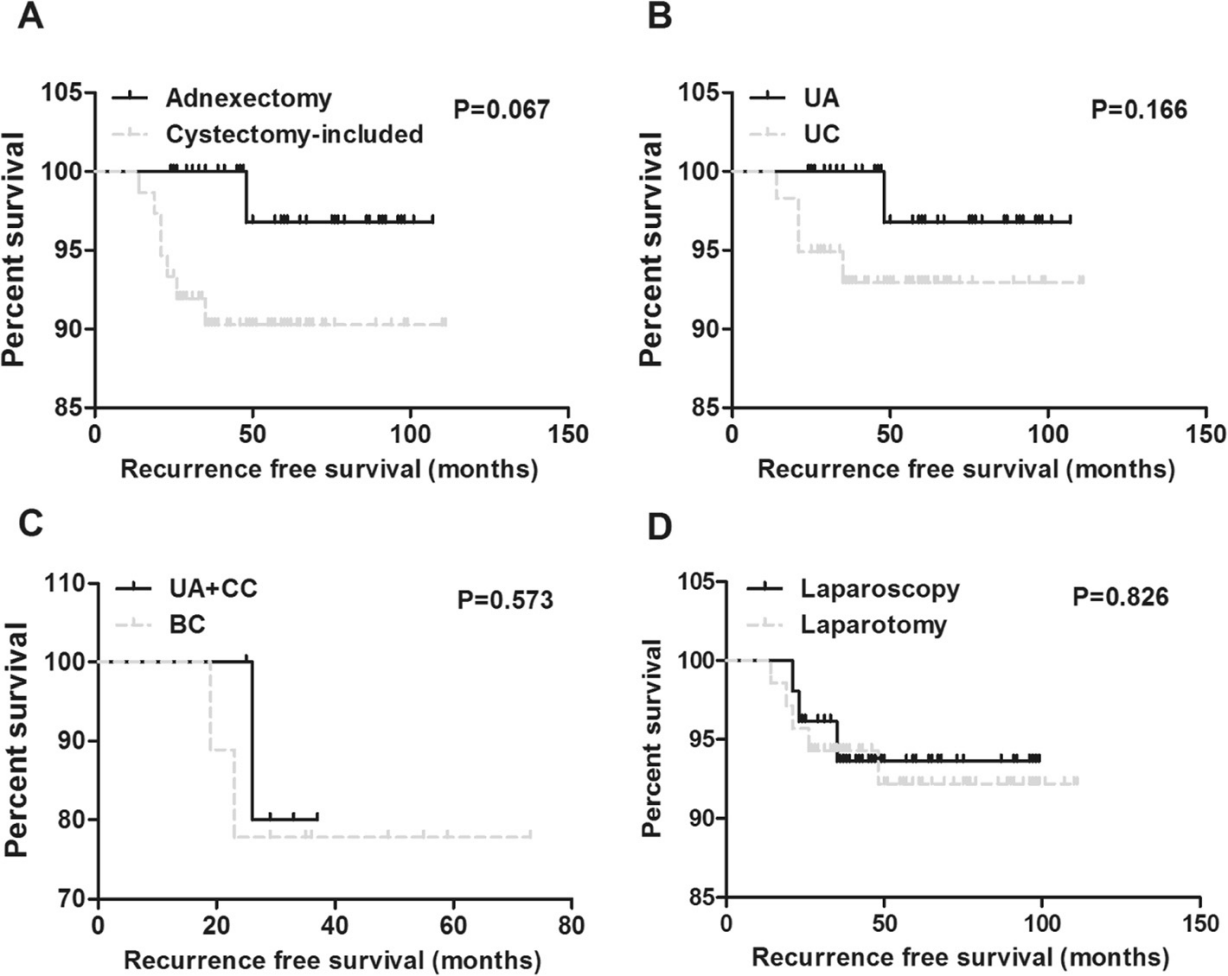
Recurrence free survival rates after fertility-sparing surgeries. **a** Comparison of recurrence free survival rates between different surgical procedures (adnexectomy and cystectomy-included). **b** Comparison of recurrence free survival rates in unilateral BOT patients(UA and UC). **c** Comparison of recurrence free survival rates in bilateral BOT patients (UA + CC and BC). **d** Comparison of recurrence free survival rates between different surgical approaches (laparotomy and laparoscopy)

## Methods

The retrospective study enrolled 122 patients with BOTs who underwent fertility-sparing surgery from Jan. 1^st^ 2003 to Dec. 31^st^ 2010 in Obstetrics and Gynecology Hospital of Fudan University. Information was collected by reviewing medical files, making systematic phone calls or performing out-patient clinic interviews. Recurrence and pregnancy information were collected after at least 2 years’ follow-up (until December 31^st^, 2012). Patients were scheduled for a pelvic examination, pelvic ultrasound examination (CT or MRI scan if necessary), and CA125 measurement every 3 months during the first year after surgery, and then every 6 months for 2 years and then annually follow up.

In addition to tumor’s FIGO stage and pathology type, whether a BOTs patient should receive conservative treatment depended on the patient’s age, wish to preserve fertility, prior surgeries received and general health conditions. These criteria were fully evaluated by experienced gynecologic oncologists before surgery. Fertility-sparing surgery was defined as a procedure that preserved the uterus and at least part of one ovary with the goal of fertility preservation. Histological typing of tumor was performed according to the WHO 2003 system, and tumor staging system was based on the FIGO 2006 system. All tumor specimens were reviewed by at least two experienced pathology specialists.

All statistical analyses were conducted with the Statistical Package for Social Sciences software, version 16.0 (SPSS 16.0, Chicago, IL, USA). Categorical variables were assessed using the Chi-squared test or the Fisher exact test, and quantitative variables were compared using Student’s T-test or the Mann-Whitney U-test. The survival curves were drawn using the Kaplan-Meier method, and significant differences between the curves were assessed with the long-rank test. A *p* value of <0.05 was considered to be statistically significant.

## Results

### Patient characteristics

A total of 122 patients with BOTs underwent conservative surgery from Jan. 1^st^ 2003 to Dec. 31^st^ 2010 in the Obstetrics and Gynecology Hospital of Fudan University were reviewed in this study. The demographic and clinicopathologic characteristics of enrolled patients were shown in Table 1. The median age of these young patients was 30.2 years (range, 11–49 years) and 79 patients (64.8 %) were nulliparous. The serum level of CA125 was tested preoperatively in 83 patients and elevated in only 29 cases (34.9 %). Majority of patients (*n* = 87) complained abdominal masses for their first visit, then abdominal pain and enlarged abdominal perimeters. There were 111 patients diagnosed at early stage, of them 70.5 % cases (*n* = 86) were at stage Ia. The average diameter of BOTs was 10.5 cm. Tumors were limited inner-ovary in 94 patients, while extra-ovary in the other 28 cases. BOTs were unilateral in 86.9 % patients (*n* = 106) and the most common pathology type was serous (SBOTs) (*n* = 64, 52.5 %), then mucinous (MBOTs) (*n* = 51, 41.8 %).

Univariate analysis showed that bilateral tumor associates with higher recurrence rate in compare with that of unilateral tumor (18.7 % vs. 6.6 %, p = 0.07). Patients with elevated CA125, bilateral tumors, extra-ovary tumor or mucinous type tended to replase in shorter time (p < 0.05)

**Table 1:** The demographic and clinicopathological characteristics of patients with BOTs

	Totality (*n*, %)	No recurrence (*n*, %)	Recurrence (*n*, %)	*P* value	Recurrence interval (m, mean)	*P* value	Infertility (*n*, %)	Pregnancy (*n*, %)	*P* value
Total number	122	114 (93.4 %)	8 (6.6 %)	N/A	25.9		11 (24.4 %)	34 (75.6 %)	
Age (y, mean ± SD)	30.2 ± 7.3	30.3 ± 7.3	29.1 ± 6.8	0.65	N/A		29.7 ± 4.4	27.6 ± 4.5	0.19
Parity									
Multiparous	43 (35.2 %)	N/A			N/A		N/A		
Nulliparous	79 (64.8 %)								
Tumor marker CA125 (tested)	83								
Normal	54 (65.1 %)	50 (92.6 %)	4 (7.4 %)	0.69	25.7	0.00	N/A		
Elevated	29 (34.9 %)	26 (89.7 %)	3 (10.3 %)		22.4				
Lesion location									
Inner-ovary	94 (77.0 %)	89 (94.7 %)	5 (5.3 %)	0.38	31.9	0.00	6 (18.8 %)	26 (81.2 %)	0.25
Extra-ovary	28 (23.0 %)	25 (89.3 %)	3 (10.7 %)		22.1		5 (38.5 %)	8 (61.5 %)	
Lesion size (cm ± SD)	10.5 ± 7.0	10.4 ± 7.0	12.0 ± 7.9	0.59	N/A		8.7 ± 4.7	10.1 ± 6.8	0.52
Lesion lateral									
Unilateral	106 (86.9 %)	101 (95.3 %)	5 (4.7 %)	0.07	33.2	0.00	8 (22.9 %)	27 (77.1 %)	0.69
Bilateral	16 (13.1 %)	13 (81.3 %)	3 (18.7 %)		23.0		3 (30.0 %)	7 (70.0 %)	
FIGO stage									
Stage I	111 (91.0 %)	104 (93.7 %)	7 (6.3 %)	0.54	30.4	N/A	8 (20.0 %)	32 (80.0 %)	0.09
> = Stage II	11 (9.0 %)	10 (90.9 %)	1 (9.1 %)		26.0		3 (60.0 %)	2 (40.0 %)	
Pathology types									
Endometriod	5 (4.1 %)	4 (80.0 %)	1 (20.0 %)	0.29	21.0	N/A	0	3 (100 %)	0.57
Serous and/or mucious	117 (95.9 %)	110 (94.0 %)	7 (6.0 %)		30.8		11 (26.2 %)	31 (73.8 %)	
Serous	64 (52.5 %)	60 (93.8 %)	4 (6.2 %)	1.00	35.9	0.00	9 (32.1 %)	19 (67.9 %)	0.45
Mucinous	51 (41.8 %)	48 (94.1 %)	3 (5.9 %)		18.5		2 (15.4 %)	11 (84.6 %)	
Serous and mucinous	2 (1.6 %)	2	0	N/A			0	1	N/A

*Abbreviations*: *BOT* borderline ovarian tumor FIGO, the International Federation of Gynecology and Obstetrics, *N/A* not applicable

### Fertility-sparing surgery

#### Adnexectomy & cystectomy

Along with the development of minimally invasive surgical techniques, National Comprehensive Cancer Network (NCCN) kept updating the guidelines for BOTs patients’ management, and we also followed the updates in our clinical practice in our hospital (as showed in Tables 2 and 3). Until the end of our study, the average observation period was 65.1 months in adnexectomy group and 53.4 months in cystectomy-included groups, respectively. It indicated that young women with BOTs tended to choose even more conservative treatment in recent years Compared with adnexectomy group, the recurrence rate was higher and the recurrence interval was shorter in cystectomy-included group, though the difference did not reach statistical significance (9.3 % vs 2.1 % *p* = 0.15, 48.0 months vs 22.7 months, N/A)(as shown in Fig. 1). Our study showed that only one patient progressed to invasive cancer but none died within our observation period. On the other hand, both surgical strategies provide good subsequent pregnancy rate (up to 75.0 %) for the BOTs patients. Until the end of our study, there was no abnormal fetus delivered.

**Table 2:** Recurrence and pregnancy outcomes in women with BOTs after fertility-sparing surgery

	Totality (*n*, %)	No recurrence (*n*, %)	Recurrence (*n*, %)	*P* value	Recurrence interval (m, mean)	*P* value	Infertility (*n*, %)	Pregnancy (*n*, *%*)	*P* value
Total number	122	114 (93.4 %)	8 (6.6 %)	N/A	25.9		11 (24.4 %)	34 (75.6 %)	
Surgical procedures									
Adnexectomy	47	46 (97.9 %)	1 (2.1 %)	0.15	48.0	N/A	3 (23.1 %)	10 (76.9 %)	1.00
Cystectomy inclued	75	68 (91.7 %)	7 (9.3 %)		22.7		8 (25.0 %)	24 (75.0 %)	
Staging surgery									
No	84 (68.9 %)	78 (92.9 %)	6 (7.1 %)	1.00	32.1	N/A	6 (18.7 %)	26 (81.3 %)	0.08
Yes	38 (31.1 %)	36 (94.7 %)	2 (5.3 %)		21.0		6 (46.2 %)	7 (53.8 %)	
Adjuvant chemotherapy				0.58					1.00
Yes	12 (9.8 %)	11 (91.7 %)	1 (8.3 %)		26.0	N/A	2 (25.0 %)	6 (75 %)	
No	110 (90.2 %)	103 (93.6 %)	7 (6.4 %)		30.4		9 (24.3 %)	28 (75.7 %)	

Cystectomy-inclued group included UC,BC,UA + CC

*Abbreviations*: *N/A* not applicable

**Table 3:** The operation situation and results of recurrence and pregnancy in different fertility-sparing surgery groups

Clinical characteristics	Surgical procedure		*p* value	Surgical procedure		*p* value	Surgical approach		*p* value
	UA	UC		UA + CC	BC		Laparotomy	Laparocsopy	
Total number (*n*)	47	59		7	9		70	52	
Operation duration (min, mean ± SD)	106.8 ± 49.8	90.6 ± 38.8	0.06	194.6 ± 70.4	165.5 ± 82.0	0.46	104.7 ± 47.4	113.3 ± 66.8	0.43
Intro-operation blood loss (ml, mean ± SD)	91.7 ± 85.1	105.7 ± 152.8	0.58	202.9 ± 139.8	144.4 ± 72.6	0.19	124.9 ± 151.9	86.7 ± 76.6	0.07
Hospitalization days (days, mean ± SD)	10.5 ± 4.2	9.6 ± 3.5	0.19	14.7 ± 5.6	14.8 ± 5.7	0.98	11.8 ± 4.5	9.1 ± 3.6	0.00
Days of antibiotic using (days, mean ± SD)	3.2 ± 1.1	3.2 ± 0.9	0.8	4.1 ± 0.9	3.1 ± 0.3	0.01	3.4 ± 1.0	3.0 ± 0.7	0.01
Menstrual status (*n*, %)									
Regular	38 (80.9 %)	47 (79.7 %)	0.88	4 (57.1 %)	7 (77.8 %)	1.00	55 (78.6 %)	41 (78.8 %)	0.97
Irregular	9 (19.1 %)	12 (20.3 %)		3 (42.9 %)	2 (22.2 %)		15 (21.4 %)	11 (21.2 %)	
Climacteric symptom (*n*, %)	5 (10.6 %)	4 (6.8 %)	0.51	2 (28.6 %)	0	0.18	8 (11.4 %)	3 (5.8 %)	0.35
Relapse (*n*, %)	1 (2.1 %)	4 (6.8 %)	0.38	1 (14.3 %)	2 (22.2 %)	1.00	5 (7.1 %)	3 (5.8 %)	1.00
Recurrence interval (m, mean ± SD)	48.0	22.8 ± 8.8	N/A	26.0	21.0	N/A	25.5 ± 15.3	26.3 ± 7.6	0.92
Invasive recurrence (*n*)	0	1 (1.7 %)	N/A	0	0	N/A	1 (1.4 %)	0	N/A
Disease related death (*n*)	0	0	N/A	0	0	N/A	0	0	N/A
Patients desired to get pregnancy (*n*, %)	13	22		3	7		27	18	
Pregnancy	10 (76.9 %)	17 (77.3 %)	1.00	2 (66.7 %)	5 (71.4 %)	0.57	18 (66.7 %)	16 (88.9 %)	0.16
Inferterlity	3 (23.1 %)	5 (22.7 %)		1 (33.3 %)	2 (28.6 %)		9 (33.3 %)	2 (11.1 %)	

*Abbrevations*: *UA* unilateral adnexectomy, *UC* unilateral cystectomy, *UA + CC* unilateral adnexectomy + contralateral cystectomy, *BC* bilateral cystectomy

*N/A* not applicable

### Unilateral adnexectomy vs Unilateral cystectomy

There were 106 cases with unilateral BOTs in this study, 47 women underwent UA and 59 underwent UC. The operation duration was shorter in the UC group than in the UA group (*p* = 0.06). However, more patients replased in shorter time in UC group, though this difference was not statistically significant (6.8 % vs 2.1 %, *p* > 0.05, 48.0 vs 22.8 months, N/A) (as shown in Fig. 1). The operational blood loss, total duration of hospitalization and days of antibiotic use were similar (*p* > 0.05), and 85 patients in these two groups still had regular menstruation after treatment. Twenty-seven pregnancies were achieved in 35 cases with unilateral BOTs, including 10 (76.9 %) in the UA group and 17 (77.3 %) in the UC group.

### Unilateral adnexectomy + contralateral cystectomy vs Bilateral cystectomy

Among 16 patients with bilateral BOTs, seven underwent UA + CC, and nine underwent BC. The operation durations, hospitalization days and operational blood loss were similar between the two groups, but the days of antibiotic using were significantly shorter in BC group (*p* < 0.05). Compared to cases with unilateral BOTs, the recurrence rate was higher and the replase interval was shorter in patients with bilateral tumors, especially in BC group. Though the difference was not statistically significant between the BC and UC + CC groups (22.2 % vs 14.3 %,21 vs 26 months, *p* > 0.05)(as shown in Fig. 1). Patients in UA + CC group were more likely to have irregular menstruation and climacteric symptoms after surgery Accordingly, the subsequent fertility rates were 66.7 % (2/3) in UA + CC group and 71.4 % (5/7) in BC group, respectively. However, the overall pregnancy rate was still satisfying in patients with bilateral tumors after conservative surgery.

### Laparoscopy vs Laparotomy

In this study of 122 cases with BOTs, 52 patients underwent laparoscopy, and 70 patients underwent laparotomy. Though the operation duration was similar between two groups, the operational blood loss, duration of hospitalization and days of antibiotic using were much lower in laparoscopy group. As for recurrence results, three of 52 patients in laparoscopy group and five of 70 patients in laparotomy group (5.8 % vs 7.1 %, *p* > 0.05) were relapsed in average 26.3 and 25.5 months respectively (as shown in Fig.1). What’s more, the status of menstruation after treatment was similar between these two groups. However, 11.1 % cases were confronted with infertility in laparoscopy group and that rate was up to 33.33 % in the laparotomy group, indicating a tendency of higher infertility rate in the latter group, but the difference was not statistically significant (*P* > 0.05).

### Staging surgery

In our study, 38 patients underwent staging surgery, while the remaining 84 patients did not. As for the specific staging procedures, omentum resection/biopsy, appendectomy, lymph node dissection/biopsy, peritoneal biopsy and contralateral ovary biopsy were performed in 18, 7, 7, 9, and 19 patients, respectively. The pathological positive results were only found in one patient done by omentum resection and two patients done by lymph node dissection. Though staging surgery to some extend advanced the FIGO stage, the recurrence results wasn’t improved much by it (*P* > 0.05). Two patients (5.3 %) in the staging surgery group experienced disease relapse in 21.0 months, while six patients (7.1 %) in 32.1 months in the non-staging surgery group. As for fertility preservation, the fertility rate was 53.8 % in the staging surgery group and that rate ran up to 81.3 % in the non-staging group. Though the difference was not statistically significant (*p* > 0.05), it still indicated an obvious declination of fertility ability in staging surgery group.

### Adjuvant chemotherapy

Most patients in our study hadn’t received chemotherapy after conservative treatment. Only 12 patients underwent adjuvant chemotherapy and most of them were in late FIGO stage (≥Ic) or with invasive implants. But the results showed that they didn’t seem to get any benefit from it both in recurrence and pregnancy. There were 8.3 % patients, who received adjuvant chemotherapy, got disease recurred after 26.0 months. Accordingly, 6.4 % cases without chemotherapy had disease recurrence in 30.4 months after conservative treatment. What’s more, the fertility outcomes were nearly the same in patients with or without adjuvant chemotherapy and both of them were about 75.0 % successful pregnancy rate.

## Discussion

As long-term survival rates of ovarian cancer patients improved and advanced assisted reproductive technology developed, there is increased interest in fertility-sparing surgery among young women with ovarian tumors [10, 11]. In literatures, the recurrence rate of BOTs varies between 0 and 25 %, with a 1–3 % invasive relapse rate [12, 13]. A systematic review [14] of 923 patients from 19 studies calculated that the recurrence rate was 16 %, with five disease-related deaths, and a subsequent pregnancy rate of 48 % after fertility sparing treatment. On the other hand, Silva EG group [15] reported the exist of late recurrence in BOTs, which showed that recurrence rate were 10 %, 19 %, 10 % and 5 % in 5 years, 10 years, 15 years and more than 15 years, respectively. These clinical characteristics provide a good opportunity for younger patients with BOTs to give birth after conservative treatments [16]. However, the invasive recurrence and disease-related deaths did occur and the pregnancy rates varied greatly among different studies. Emile Dara reported [17] the subsequent pregnancy rates decreased to 34 % and the risk of recurrence and lethal recurrence accordingly increased to 38 % and 2 % in advanced stage BOTs. Therefore, retrospective analyses of recurrence and fertility related factors may help to develop better clinical strategies for young women with BOTs, especially when mentioned the specific fertility-sparing surgical procedure.

Cystectomy is always supposed to preserve more normal ovarian tissue and therefore increase pregnancy rates after conservative surgery. However the oncological safety and true fertility benefits are still unknown. In our study, we compared the recurrence rates and pregnancy outcomes of four fertility-sparing surgeries (UA, UC, UA + CC, and BC). Compared to UA group, the recurrence rate in cystectomy-included group increased from 2.1 % to 9.3 % and the relapse interval decreased from 48.0 months to 22.7 months. The tendency was even clear in the BC group (22.2 %, 21.2 months). These data aligned with the results from most published literatures. As for patients with unilateral BOTs, the fertility rates were satisfying in both UA and UC groups in our study (76.9 % vs 77.3 %). Even in cases with bilateral BOTs, the pregnancy outcomes were still encouraging in both UA + CC and BC groups (71.4 % vs 66.7 %). But BC group was accompanied by an enhanced recurrence rate (22.2 % vs 14.3 %). However, there are different opinions regarding the conservative treatment of bilateral BOTs. Researchers [18] enrolled 32 young women with bilateral BOTS in a randomized controlled study. And they found that the cumulative pregnancy rate and the cumulative probability of first pregnancy were significantly higher in the BC group compared to UA + CC group (14/15 vs 9/17; 5 months vs 8 months). However, no negative effects were observed in the cumulative probability of first recurrence in BC group (9/15 vs 10/17). A possible reason for this finding may be that all 32 BOT patients in that group underwent radical surgery in a short time after fertility finished. Therefore, adnexectomy surgery should be the first choice in unilateral BOT patients. However, for patients with bilateral ovarian tumors or with a history of previous ovarian surgery, UC or BC could still be considered for those with a strong desire for childbearing in short term, with the premise of fully informed of easier relapse.

In recent years, laparoscopy was thought to be a minimally invasive surgical procedure, which could better protect normal ovarian tissue and reduce pelvic adhesion. These features satisfy the main objectivity of conservative surgery. Thus, laparoscopy has been gradually preferred by young patients with ovarian tumors. Researchers have found that tumor rupture rate was higher in laparoscopy group [19]. However, it has been inconclusive whether high tumor rupture rate was an independent adverse prognostic factor for recurrence. In some published studies, it was indicated that an increased tumor rupture rate enhanced the recurrence rate after surgery [20]. However, the conclusions were different in other researches. Ødegaard E [21] retrospectively analyzed 107 patients with BOTs. They observed that the rate of intra-operative tumor rupture wasn’t significantly elevated during laparoscopy if only when tumor diameter exceeded 10 cm, and it did not have a negative impact on prognosis and pregnancy rate. Our study had drawn a similar conclusion that, compared to laparotomy, laparoscopy had no disadvantage in terms of recurrence results. But it sharply reduced the risk of infertility from 33.1 % to 11.1 % after conservative surgery. All these researches indicated that patients with short-term fertility plans could be recommended for laparoscopy and pockets should be used to extract the tumor tissues during surgery.

Though fertility-sparing surgery has been generally accepted as a treatment for young patients, there is less agreement on staging surgery in BOTs. Trillsch F et al [22] had evaluated the risk of staging procedures in 559 patients with serous BOTs. And they concluded that the recurrence risk significantly increased if more than two steps were skipped and the most crucial procedure was omentectomy (HR 1.91). In some other reports, researchers have also confirmed the association between incomplete staging surgery and shorter recurrence intervals [23]. However, even more researchers thought that complete staging may elevate FIGO stage but provide no benefit in reducing recurrence rate and improving overall survival [24]. Kristensen GS [25] had evaluated the sensitivity and specificity of random biopsies, omentectomy, and hysterectomy; then he denied the benefits of staging surgery in BOTs with a macroscopically normal appearance. This finding were in accord with the studies performed by Pirimoglu ZM [26] and we draw a similar conclusion. In our observation, staging surgery had only 5 % (3/60) positive result, which increased the FIGO stage but had no effects on recurrence. However, the pregnancy rate was sharply decreased from 81.3 % to 53.8 % (*p* = 0.08). Because of the usual negative results of staging surgery and the potential adverse impact on the fertility, patients with apparent early stage BOTs were not always recommended for surgical staging in fertility sparing surgery. Whether or not to take adjuvant chemotherapy in patients with BOTs has also been discussed for a long time. Alexandra Leary [27] had reported on 36 patients with invasive implants serous BOTs and they were treated with surgery and platinum-based chemotherapy. Among these patients, 13 (36 %) relapsed, and eight patients (22.2 %) progressed to invasive disease. However, the 5-year progressive free survival (PFS) and overall survival (OS) were improved, which were 67 % and 96 % respectively. But there were some different observations in our study. There were only 12 patients, who were at advanced stages or with invasive implants, received chemotherapy after surgery. The recurrence rate was 8.3 % and the pregnancy rate was 75 %, which indicated that the patients did not receive any benefit in recurrence and fertility from chemotherapy. This finding was consistent with some other published literatures [28, 29]. Ren J [30] had studied 64 patients underwent postoperative chemotherapy and concluded that there was no significant difference in recurrence and overall survival rates. Even for patients with invasive implants, some studies still had not demonstrated any positive result [31]. So we would carefully conclude that for BOT patients with a strong childbearing desire, adjuvant chemotherapy should be avoided for the shortest pregnancy planning period.

## Conclusion

In conclusion, conservative surgery is relative safe and beneficial for young women with BOTs who have a strong desire for preserving their fertility ability. For most patients with unilateral tumor, laparoscopic unilateral adnexectomy should be the first choice because of its low recurrence rate and high successful fertility rate. However, patients with bilateral tumors, elevated CA125, extra-ovary tumor or mucinous type are tended to recurrence in shorter interval, conservative treatment should be carefully advised and pregnancy should be achieved as soon as possible after fertility sparing surgery. Moreover, Cystectomy-included surgeries are still acceptable for patients with bilateral BOTs or those who had undergone ovarian surgery before. So far, there was no enough evidence to show benefit of surgical staging in recurrence outcomes. But the invasive staging procedures may lead to higher infertility rate. The staging procedures often just advance the clinical stage, not improve the prognosis. Most experts recommend less aggressive surgery and comprehensive staging. Thus, surgical staging is not always necessary in fertility sparing surgery, especially for early stage BOTs. In addition, the benefits of postoperative chemotherapy have not been demonstrated for BOT patients without invasive implants, both in previous reports and our current study. This relatively large group study demonstrated that fertility sparing surgery in younger patients with BOTs is feasible. Long time follow up, overall survival rate and impact to next generation need to be further investigated in future. Overall, patients with BOTs should be fully assessed by experienced gynecological oncologist, and well informed about the surgical risks and benefits before fertility sparing surgery. What’s more, the patients should follow up closely and for long term after conservative surgery.

### Ethics approval and consent to participate

Obstetrics and Gynecology Hospital of Fudan University Human Research Ethics Committee approval was obtained for the use of all samples (reference number [2015] 43).

### Consent for publication

Not applicable.

## Abbreviations

BC: bilateral cystectomy
BOTs: borderline ovarian tumors
FIGO: International Federation of Gynecology and Obstetrics
MBOTs: mucinous borderline ovarian tumors
N/A: not applicable
OS: overall survavil
PFS: progressive free survival
SBOTs: serous borderline ovarian tumors
UA: unilateral adnexectomy
UA + CC: unilateral adnexectomy + contralateral cystectomy
UC: unilateral cystectomy
WHO: World Health Organization
